# Endobronchial Ultrasound under Moderate Sedation versus General Anesthesia

**DOI:** 10.3390/jcm7110421

**Published:** 2018-11-08

**Authors:** Maria Gabriela O. Fernandes, Vanessa F. Santos, Natália Martins, Maria C. Sucena, Madalena M. Passos, Maria Manuel Marques, Adriana M. Magalhães, António Bugalho

**Affiliations:** 1 Department of Pulmonology, Centro Hospitalar de São João, Alameda Professor Hernâni Monteiro, 4200-319 Porto, Portugal; vferreirads@gmail.com (V.F.S.); ncmartins@med.up.pt (N.M.); maria.sucena@hotmail.com (M.C.S.); adrimagalhaes08@gmail.com (A.M.M.); 2 Faculty of Medicine, University of Porto, Alameda Professor Hernâni Monteiro, 4200-319 Porto, Portugal; 3 Institute for Research and Innovation in Health (I3S), University of Porto, Rua Alfredo Allen, 4200-135 Porto, Portugal; 4 Department of Anaesthesiology, Centro Hospitalar de São João, Alameda Professor Hernâni Monteiro, 4200-319 Porto, Portugal; madalena_m_passos@hotmail.com; 5 Department of Anaesthesiology, Hospital Beatriz Ângelo, Avenida Carlos Teixeira, 3, 2674-514 Loures, Portugal; mm.marques@hbeatrizangelo.pt; 6 Department of Pulmonology, Hospital Beatriz Ângelo, Avenida Carlos Teixeira, 3, 2674-514 Loures, Portugal; antonio.bugalho@jmellosaude.pt; 7 Department of Pulmonology, CUF Infante Santo Hospital and CUF Descobertas Hospital, 1350-070 Lisboa, Portugal

**Keywords:** endobronchial ultrasound, general anesthesia, sedation, diagnosis, staging

## Abstract

Background: Different anesthetic protocols may influence endobronchial ultrasound-guided needle aspiration (EBUS-TBNA) outcomes, patient comfort, and even safety. In this study, two anesthesia techniques were assessed and compared for EBUS-TBNA. Methods: A prospective, multicenter study was carried out. Patients were allocated to Group 1 (general anesthesia with neuromuscular blockade and controlled ventilation) and Group 2 (intravenous sedation). EBUS-TBNA accuracy was the primary outcome. Safety, patient comfort and satisfaction, and operators’ difficulties were defined as secondary outcomes. Results: Of the 115 patients enrolled (Group 1 = 59, Group 2 = 56), EBUS-TBNA was performed for hilar or mediastinal lesion diagnosis and lung cancer staging in, respectively, 77 (67%) and 38 (33%) patients. The numbers of lymph nodes stations (1.8 ± 1.0 vs. 1.7 ± 1.0, *p* = 0.472) and punctures per station (6.9 ± 3.1 vs. 6.0 ± 2.5, *p* = 0.084) were similar between groups. Adequate samples were obtained from 109 patients (97.3%) with similar diagnostic accuracy. Procedure duration was not significantly different (*p* = 0.348). Hemodynamic parameters and systolic and diastolic blood pressures were higher in Group 1 at the beginning and at the end of the procedure. Adverse events were equally distributed, and no significant differences were found regarding patient satisfaction and bronchoscopist/anesthesiologist difficulties. Conclusions: The type of anesthesia used did not influence EBUS-TBNA outcomes. EBUS-TBNA performed under sedation or general anesthesia did not affect the diagnostic yield, complication rate, and patients’ comfort and satisfaction.

## 1. Introduction

Endobronchial ultrasound-guided needle aspiration (EBUS-TBNA) is useful in the diagnosis of a wide range of clinical situations, besides being considered the first-choice method for mediastinal staging of lung cancer due to its high yield, low rate of complications, and reduced costs [1,2]. For optimal performance, it is desirable to have a collaborative patient; the procedure induces cough, increases airway secretions, and reduces airway caliber. Furthermore, during the EBUS-TBNA staging procedure, all relevant lymph node stations should be evaluated and punctured at least three times, resulting in increased procedure length and patient discomfort [3,4].

The type of sedation used during EBUS-TBNA is also extremely important, because it can affect the diagnostic accuracy, procedure safety, and patient comfort, and at same time determines the need for additional health-care resources and increased care costs. A guideline and panel report of experts suggest that moderate or deep sedation are acceptable approaches during EBUS-TBNA [5], but the poor quality of the published studies has resulted in a weak recommendation (grade 2C) [6]. In addition, sedation protocols are quite variable across institutions and are usually based on individual parameters, local resources, and operator preferences. Initial studies were done under general anesthesia [5], but recently, several centers have started using moderate or deep sedation. In fact, studies conducted to evaluate and compare different sedation levels during EBUS-TBNA have shown conflicting results [7,8,9].

In this sense, based on the existing literature, a prospective, multicenter study was conducted to assess patient safety and comfort, EBUS-TBNA diagnostic accuracy, and related complications under two different protocols: (1) total intravenous general anesthesia with neuromuscular blockade under controlled ventilation, and (2) intravenous sedation.

## 2. Experimental Section

### 2.1. Study Design and Measured Outcomes

This prospective, multicenter study was performed between March and October 2014 in two pulmonology departments, (1) at the Centro Hospitalar de São João (Porto, Portugal), an academic tertiary institution, and (2) at the Hospital Beatriz Ângelo (Lisbon, Portugal), a non-tertiary hospital. It was approved by both hospital ethical committees, and all patients gave written informed consent before being enrolled in this study.

The study primary outcome was EBUS-TBNA accuracy, determined based on the adequate number compared to the total number of samples. Cytological samples were validated and classified by a pathologist as positive (providing a diagnosis of cancer or specific benign disease), adequate (when lymph nodes presented a suitable cellular component, lymphocytes, and pigmented macrophages), or inadequate (when cellular components, blood, or bronchial epithelial cells were absent or there was insufficient material to obtain a definitive diagnosis). In the case a definitive diagnosis could not be achieved, surgical procedures or clinical and radiological follow-up for at least 12 months were recommended. As study secondary outcomes we considered safety, patient comfort and satisfaction, and operators’ difficulties. Safety was measured by the EBUS-TBNA rate and anesthetic-related complications, and by hemodynamic parameter analysis. Comfort was assessed by a specific questionnaire and by the patient´s willingness to repeat the exam. Bronchoscopist and anesthesiologist difficulties were recorded by means of a specific questionnaire.

### 2.2. Patients

All patients referred for EBUS-TBNA, aged over 18 years, and with the capacity to sign the informed consent form were included in this study. Exclusion criteria included contraindications for EBUS or tracheal intubation, history of anesthetic drug allergy, pregnancy, need for additional bronchoscopic procedures, and presence of severe neuropsychiatric conditions that affected the cognitive ability to answer questionnaires.

### 2.3. Study Protocol

**Figure 1:**
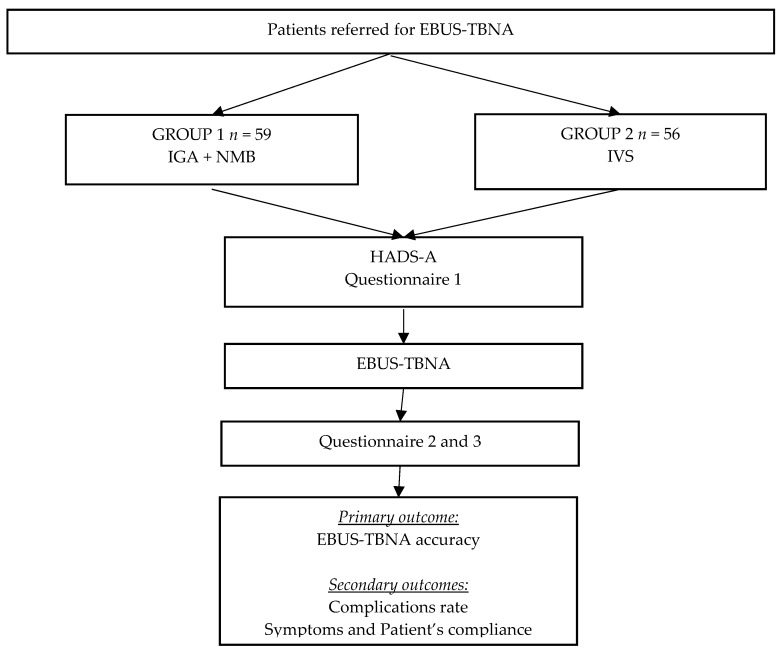
Study protocol. IGA, intravenous general anesthesia; NMB, neuromuscular blockade; IVS, intravenous sedation; EBUS-TBNA, endobronchial ultrasound-guided needle aspiration; HADS-A, Hospital Anxiety and Depression Scale.

Patients referred for EBUS-TBNA (Figure 1) were randomly allocated to Group 1 (general anesthesia with neuromuscular blockade and controlled ventilation) or Group 2 (intravenous sedation). Initial clinical assessment included demographic data, comorbidities, smoking habits and medication. Baseline anxiety status was assessed using the “Hospital Anxiety and Depression Scale” (HADS-A) [10,11], and a specific EBUS-TBNA self-assessment questionnaire on knowledge, fears, and expectations was provided.

The hemodynamic parameters of the patients (blood pressure, pulse oximetry and heart rate) were continuously monitored. Three time points of the anesthetic procedure were considered for analysis: beginning (T1), middle (T2), and end (T3). Procedure duration was measured from the beginning (T1) until the end of the anesthesia (T3). Drugs and doses used for sedation were also documented. All procedures were performed in an outpatient basis.

Before discharge, all patients included answered a second questionnaire on exam tolerance and general satisfaction, respectively, assessing the main symptoms (i.e., cough, dyspnea, pain) and the willingness to repeat EBUS-TBNA. Another specific questionnaire was also given to the bronchoscopists and anesthesiologists.

### 2.4. Anesthetic Procedure

Patients assigned into Group 1 were submitted to intravenous general anesthesia (IGA) with neuromuscular blockade (NMB) under controlled ventilation. EBUS was introduced through an artificial airway (laryngeal mask, orotracheal tube, or rigid scope). Drug options allowed were alfentanyl/fentanyl, propofol, midazolam, succinylcholine, rocuronium, and sugammadex.

Patients assigned into Group 2 were submitted to local oropharynx and larynx anesthesia with 2% lidocaine and intravenous sedation, using a combination of alfentanyl/fentanyl, propofol, and/or midazolam. EBUS was introduced through a mouthpiece and oxygen was delivered through a face mask. Patients were kept under spontaneous breathing. Sedation level was monitored with the Ramsey Sedation Scale (RSS) [12] (Appendix A Table A1), aiming for a sedation level ≥4. Time to recovery was assessed by the Aldrete score [12], where the patients were discharged after reaching an Aldrete score ≥9.

Midazolam was administered in both groups. All anesthetic procedures were performed by two experienced anesthesiologists (>10 years practice). A specific questionnaire was also applied to verify the anesthetic difficulties experienced during induction, maintenance, and recovery, and factors associated with potential problems or complications.

Regarding hemodynamic parameters, hypotension was defined as a systolic pressure <90 mmHg at any time of the procedure, while hypertension was defined as an increase (>30%) in mean arterial blood pressure in relation to baseline values, and hypoxemia as a partial pressure of SpO_2_ <90% for more than 30 s at any time in the procedure.

The presence or absence of laryngospasm/bronchospasm was determined by pulmonary auscultation and if there was need for specific treatment or bronchodilator use.

### 2.5. EBUS-TBNA Technique

EBUS-TBNA was performed as previously reported [13]. A flexible ultrasound bronchoscope (BF-UC180F, Olympus, Tokyo, Japan) with an integrated convex transducer (7.5 MHz) and Doppler mode was used. Images were manipulated using an ultrasound console (EU-ME1, Olympus, Tokyo, Japan or Prosound Alpha10, Aloka, Tokyo, Japan). Once the target lesion was identified, transbronchial punctures were done with a 22-gauge needle (NA-201SX-4022, Olympus, Tokyo, Japan). A minimum of four needle passes were done for each lymph node. Then, aspirated specimens were expelled into a container with preservative liquid, and a cytobloc was prepared and stained for subsequent cytological examination. Rapid on-site evaluation was not performed, and cytologists did not know the type of anesthesia applied during EBUS-TBNA.

EBUS-TBNA was performed by four experienced bronchologists (>4 years practice). Then, a specific questionnaire was applied, assessing experienced difficulties during examination and/or factors that eventually contributed to a premature interruption.

### 2.6. Data and Statistical Analysis

Parameters measured included patient demographics, procedure indication, number of punctured lymph node stations and aspirations per station, pathology results, procedure time, medication doses, cardiorespiratory parameters, and complications (>50 mL bronchial bleeding, pneumothorax, mediastinitis, persistent arrhythmia requiring medication, other life-threatening conditions, death), and were compared between groups. Power analysis (GPower software, version 3.1.9.3; Dusseldorf, Germany) was used to establish the effect size for subgroup analysis.

All statistical tests were performed using the Statistical Package for the Social Sciences (SPSS), version 25.0 (SPSS, Inc., Chicago, IL, USA). Results are presented as mean values and standard deviation (SD). A Kolmogorov–Smirnov test was used to assess the normality of data. Student’s *t*-test was used to determine significant differences among two different samples for normally distributed continuous variables, and Mann–Whitney test for those non-normally distributed. Chi-squared and Fisher tests were applied for categorical variables. A *p* value of <0.05 was considered statistically significant.

## 3. Results

One-hundred and fifteen patients fulfilled the inclusion criteria, and were allocated to Group 1 (*n* = 59, 51.3%) and Group 2 (*n* = 56, 48.7%). Sociodemographic and clinical characteristics are shown in Table 1. There were no significant differences between groups for gender (*p* = 0.146), age (*p* = 0.645), educational degree (*p* = 0.495), and EBUS indication. Smokers percentage was statistically higher in Group 2 (42.9% vs. 27.1%, *p* = 0.039). Most patients (70.4%) did not show pathological anxiety on the HADS-A scale; 18.3% were scored as borderline and 11.3% had psychopathology criteria, but without significant differences between groups (*p* = 0.763). Twenty-seven patients (23.5%) used psychiatric drugs, but there were no differences between groups (*p* = 0.948).

According to EBUS-TBNA specific questionnaire 1 (Table S1), the overall knowledge and fears about the exam were similar. In Groups 1 and 2, respectively, 70.7% and 66.1% of patients had never heard of EBUS (*p* = 0.596), and the major concern was the expectation of diagnosis, which was independent of the anesthetic method. A total of 76.3% (Group 1) and 71.4% (Group 2) of patients did not mention any unpleasant moments or complaints during the procedure. One-hundred and eight patients (93.9%), regardless of group, reported that they would repeat EBUS-TBNA (*p* = 0.272). On the other hand, among patients who referred not to repeat the exam, 5 (71.4%) belonged to Group 1 (Table S1).

Regarding anesthetic procedures and hemodynamic parameters, among the three time points considered (T1, T2, T3), mean systolic and diastolic blood pressures were statistically higher in Group 1 (Table 2), while no significant differences were found between groups for heart rate and oxygen saturation.

Considering Ramsey sedation scale, in all the patients enrolled a score ≥4 was achieved (Table 3, Appendix A Table A1). The mean dose used of midazolam was 1.4 mg, being significantly higher in Group 2 as compared to Group 1 (1.6 ± 0.9 mg vs. 1.2 ± 0.9 mg, *p* = 0.018). Furthermore, Group 1 required more propofol than Group 2 (389.6 ± 157.1 mg vs. 273.0 ± 151.6 mg, *p* < 0.001), while the alfentanyl doses used were similar (*p* = 0.897) (Table S2).

EBUS-TBNA indications were equally distributed between groups (Table 4). A total of 203 lesions/lymph node stations were punctured, 108 in Group 1 and 95 in Group 2. On average, more lymph nodes stations were punctured in Group 1 when compared to Group 2 (1.8 ± 1.0 vs. 1.7 ± 1.0, *p* = 0.472), with the number of punctures per exam in Group 1 also being higher (6.9 ± 3.1 vs. 6.0 ± 2.5, *p* = 0.084), but not reaching statistical significance. Moreover, no punctures were done in three patients, (two belonging to Group 1 and one to Group 2). Lymph node tissue was obtained in 109 (97.3%) samples, the aspirated material being inadequate in two cases in Group 1 and one in Group 2 (*p* = 0.742). This corresponded to a similar diagnostic accuracy: 96.5% (55/57) in Group 1 and 98.2% (54/55) in Group 2, *p* = 0.580. Procedure duration was similar in both groups (49.9 ± 14.3 vs. 47.4 ± 14.5 min, *p* = 0.348).

Table 5 shows the results obtained for final diagnostic classification. No significant differences were found between groups (*p* = 0.299).

Minor complications occurred in 11 patients (9.6%), with five belonging to Group 1 and six to Group 2. No significant differences were stated between groups (*p* = 0.683). Bronchospasm or laryngospasm requiring bronchodilators, persistent desaturation, and hypotension occurred, respectively, in 5 (45.5%), 3 (27.3%), and 3 (27.3%) patients. Mediastinitis, one of the major adverse events, occurred in a patient belonging to Group 1 (0.9%), 15 days after the procedure. This patient was sent for restaging due to an increase in the lymph node, but it proved to be the result of Surgicel^®^ (Ethicon, Johnson & Johnson Medical, Somerville, NJ, USA), being left in mediastinum after a lobectomy with lymphadenectomy.

Data obtained after bronchoscopist and anesthesiologist questionnaires were completed are shown in Table 6. Bronchoscopist did not report EBUS-associated adversities in 67% of the cases, with the difficulties being mainly related to cough (*n* = 19, 16.5%) and target lesion location (*n* = 11, 9.6%). There were with significant differences between groups (*p* < 0.001). On the other hand, anesthesiologist did not report any obstacles to the anesthetic technique in 73.9% of the procedures (*p* = 0.053).

## 4. Discussion

EBUS-TBNA performed under general anesthesia and neuromuscular blockade, as well as with moderate sedation, seems to be safe and effective. In fact, one of the major advantages in performing EBUS-TBNA under general anesthesia and neuromuscular blockade is that optimal procedure conditions can be guaranteed for bronchoscopist, contributing to better EBUS accuracy and optimal patient comfort [14]. The disadvantages are mainly related to the need for an artificial airway, mechanical ventilation and associated complications, longer procedure duration, and, possibly, higher costs [15]. Nevertheless, none of these statements has been proven. Therefore, sedation has some advantages over general anesthesia as it avoids use of an artificial airway and controlled ventilation, with a lower hemodynamic impact and shorter recovery phase [9]. In fact, according to the recent EBUS-specific and American College of Chest Physicians (ACCP) guidelines, either moderate sedation or deep sedation are acceptable [6]. In this prospective, multicenter clinical study, moderate sedation with spontaneous breathing was compared with general anesthesia under controlled ventilation, in two distinct centers, regardless of the EBUS indications.

Although differences in clinical parameters that could influence patients’ tolerance and satisfaction, anxiety levels, psychotropic drug use, education level, and concerns and previous information about the exam were not observed in the studied population, statistically significant differences were found in relation to smoking habits. In fact, there were more active smokers in the sedation group, which might have contributed to the occurrence of cough. This symptom could not be compared with the patients of Group 1, since they were under neuromuscular blockade.

Patient comfort during the exam is difficult to assess, since all patients were exposed to midazolam. Thus, to overcome this question, a questionnaire was applied regarding specific moments of the procedure, instead of more objective symptom quantification. The most frequent complaints were the waiting period immediately before and during the onset of anesthetic procedure. Patient satisfaction was assessed by the willingness to repeat EBUS, as was done already in other studies on this issue [15,16,17]. Despite being statistically similar in both groups, more patients (*n* = 5) belonging to the general anesthesia group refused EBUS repetition than in the sedation group (*n* = 2).

Patient satisfaction was achieved with both anesthesia techniques, which means that the examination can be performed under sedation without affecting patient satisfaction. Steinfort et al. [15] were the first to reflect on patient satisfaction during EBUS, and found that EBUS-TBNA performed under conscious sedation with midazolam, fentanyl, and/or propofol was associated with higher patient satisfaction, with 98% answering that they would “definitely” would return to repeat the exam if necessary. On the other hand, Sarkiss et al. [14] described EBUS under general anesthesia in more than 200 patients without finding any major complications, but no reference was made to the diagnostic yield. In this study, EBUS-TBNA accuracy was considered similar in both groups.

Yarmus et al. [7] conducted a retrospective analysis of EBUS performed in two different centers, with different anesthetic methods: deep sedation with propofol, or moderate sedation with fentanyl and midazolam, and stated that the number of lymph nodes sampled (2.2 vs. 1.4 per patient, *p* < 0.001) and the diagnostic yield under deep sedation were higher (80% vs. 66%, *p* < 0.001) than in the moderate sedation group. Oztas et al. [18] retrospectively compared two types of sedation: propofol–midazolam and midazolam alone, and found no differences in the EBUS-TBNA diagnostic value and complication rate. Similarly, Casal et al. [8] randomly included 140 patients to undergo the procedure under general anesthesia or moderate sedation (midazolam plus fentanyl), to determine and compare the diagnostic yield, complication rate, and patient tolerance between groups. Interestingly, in our study, although there are some differences in the protocol used for anesthesia, it corroborates its findings.

The mean number of lymph nodes/lesions sampled per patient (1.8 ± 1.0) did not reach statistical difference between groups, but this value was lower in comparison with those reported in other studies, and can be explained by the predominance of the diagnostic indication over staging. In fact, EBUS was performed for lung cancer staging only in 33% of patients. In addition, a shorter procedure time would be expected in the sedation group. However, while it was somewhat shorter, the difference was not significant, as found by Postelnicu et al. [19]. Furthermore, Yarmus et al. [7] found a significantly shorter procedure time in the deep sedation group when compared to the moderate sedation group, in which more punctures were done per patient. Indeed, procedure time may also be influenced by other factors, such as the experience of the bronchoscopist or number of punctures, rather than the type of anesthesia used.

Another important and often neglected issue that should be highlighted as it alters the overall outcomes is operator preferences and skills, as mentioned by Kang et al. [20]. There were no differences in the difficulties of the procedure, as assessed by the bronchoscopists, anesthesiologists, and nurses. Similarly, in both groups, sedation was not deep enough to cause discomfort during the procedure, clearly indicating that there is no preference for sedation or general anesthesia techniques. EBUS-TBNA is recognized as being a safe procedure. In the largest published survey, including 7345 EBUS-TBNA procedures, there were sparse complications, with 14 cases (0.19%) of infectious complications and seven of mediastinitis [21]. In a multicenter registry (the Quality Improvement Registry, Evaluation and Education (AQuIRE) survey), among the 1317 patients included, 19 (1.44%; 95% confidence interval (CI), 0.87–2.24%) had EBUS-TBNA complications and one patient died. As only 24 h of complications were recorded, some cases of infection could have been lost [22]. In this study, only one serious complication was reported. Mediastinitis occurred in a patient that had previously undergone lymphadenectomy, in which Surgicel^®^ was left, but after surgical and antibiotic treatment, the patient recovered completely. In the general anesthesia group, a higher mean arterial pressure was found, probably as a consequence of endotracheal intubation. This raise may represent an increased risk for cardiovascular events, although none occurred in this study. As expected, the propofol dose was higher in the general anesthesia group, while the dose of midazolam was higher in the sedation group, and no major drug-related adverse events were observed.

## 5. Conclusions

Overall, this study, conducted in two different centers with distinct differentiation levels, clearly reflects the independence of the operator and center results. Moreover, after considering the conflicting results from retrospective studies, our results are consistent with those obtained in other randomly assigned studies. The type of anesthesia did not influence EBUS-TBNA outcomes. Thus, based on our findings it seems feasible to conclude that EBUS under moderate sedation may be the first-choice method without compromising diagnostic yield, complication rate, patient comfort, and satisfaction.

## Figures and Tables

**Table 1:** Baseline patient characteristics.

		Total	Group 1	Group 2	*p* Value
Age-years (mean ± SD)		60.1 ± 12.8	60.6 ± 13.1	59.5 ± 12.6	0.645
Gender, *n* (%)	Female	34 (29.6)	21 (35.6)	13 (23.2)	0.146
	Male	81 (70.4)	38 (64.4)	43 (76.8)	
Smoking habits, *n* (%)	Smoker	40 (34.8)	16 (27.1)	24 (42.9)	0.039
	Ex-smoker	33 (33.0)	15 (25.4)	18 (32.1)	
	Non-smoker	42 (42.0)	28 (47.5)	25 (25.0)	
Scholarity years, *n* (%)	<4	31 (27.0)	18 (30.5)	13 (23.2)	0.495
	4–9	55 (47.8)	25 (42.4)	30 (53.6)	
	9–12	15 (13.0)	7 (11.9)	8 (14.3)	
	>12	14 (12.2)	9 (15.3)	5 (8.9)	
HADS-A, *n* (%)	Psycopathology	13 (11.3)	8 (13.6)	5 (8.9)	0.763
	Borderline	21 (18.3)	7 (11.9)	14 (25.0)	
	No psycopathology	81 (70.4)	44 (74.6)	37 (66.1)	
Psychotropics, *n* (%)	Yes	27 (23.5)	14 (23.7)	13 (23.2)	0.948
	No	81 (70.4)	45 (76.3)	43 (76.8)	

HADS-A: Hospital Anxiety and Depression Scale.

**Table 2:** Hemodynamic parameters.

	Group 1	Group 2	*p* Value
Systolic blood pressure (mmHg)			
Beginning (T1)	143.5 ± 25.9	129.0 ± 21.8	0.002
Middle (T2)	114.7 ± 21.9	106.4 ± 15.0	0.020
End (T3)	127.0 ± 21.9	108.9 ± 17.0	<0.001
Diastolic blood pressure (mmHg)			
Beginning (T1)	79.4 ± 13.9	74.6 ± 12.5	0.056
Middle (T2)	69.0 ± 13.1	62.6 ± 11.0	0.005
End (T3)	74.2 ± 13.2	64.2 ± 11.9	<0.001
Heart rate (beats/min)			
Beginning (T1)	76.5 ± 17.4	73.6 ± 14.9	0.344
Middle (T2)	79.7 ± 18.2	75.9 ± 14.7	0.233
End (T3)	79.4 ± 18.4	75.1 ± 12.3	0.150
Partial oxygen pressure (%)			
Beginning (T1)	96.3 ± 2.7	95.6 ± 3.5	0.289
Middle (T2)	97.5 ± 6.2	96.5 ± 4.2	0.321
End (T3)	96.8 ± 6.4	96.5 ± 4.2	0.357

**Table 3:** Ramsey sedation scores in the studied groups.

Ramsey Scale	Group 1 (*n*)	Group 2 (*n*)
Ramsey 4	0	50
Ramsey 5	0	6
Ramsey 6	59	0

**Table 4:** EBUS-TBNA technical information.

		Total	Group 1	Group 2	*p* Value
Indication (%)	Lung cancer staging	38 (33.0)	18 (30.5)	20 (35.7)	0.553
	Diagnosis	77 (67.0)	41 (69.5)	36 (64.3)	
Procedure time (mean ± SD)		48.7 ± 14.4	49.9 ± 14.3	47.4 ± 14.5	0.348
Lymph-node stations sampled (mean ± SD)		1.8 ± 1.0	1.8 ± 1.0	1.7 ± 1.0	0.472
Punctures per exam (mean ± SD)		6.4 ± 2.8	6.9 ± 3.1	6.0 ± 2.5	0.084

**Table 5:** EBUS-TBNA diagnosis.

	Total	Group 1	Group 2	*p* Value
Malignancy *n* (%)	53 (48.6)	22 (40.0)	31 (57.4)	0.299
Granulomatous disease *n* (%)	9 (8.2)	6 (10.9)	3 (5.6)	
Lymphoproliferative disease *n* (%)	3 (2.8)	2 (3.6)	1 (1.8)	
Reactive lymph node/Negative for malignancy *n* (%)	44 (40.4)	25 (45.5)	19 (35.2)	

TBNA was not performed in three cases: two in Group 1 and one in Group 2.

**Table 6:** Bronchoscopist and anesthesiologist questionnaire.

Bronchoscopist Questionnaire				
	Total *n* (%)	Group 1 *n* (%)	Group 2 *n* (%)	*p* Value
1. In which of the following steps did you feel difficulty?				
EBUS introduction	6 (5.2)	0	6 (10.7)	<0.001
Target ultrasound identification/recognition	1 (0.9)	0	1 (1.8)	
TBNA	16 (13.9)	2 (3.4)	14 (25.0)	
Other	15 (13.0)	4 (6.8)	11 (19.6)	
None	77 (67.0)	53 (89.8)	24 (42.9)	
2. Which factors contributed to the above difficulties?				
Patient’s movement	4 (3.5)	0	4 (7.1)	<0.001
Cough	19 (16.5)	1 (1.7)	18 (32.1)	
Anatomic factors related to the target lesions	10 (8.7)	1 (1.7)	9 (16.1)	
None	82 (71.3)	57 (96.6)	25 (44.6)	
**Anesthesiologist Questionnaire**				
1. In which of the following steps did you feel difficulty?				
Induction	4 (3.6)	0	4 (7.4)	<0.001
Maintenance	16 (14.3)	2 (3.4)	14 (25.9)	
Recovery	8 (7.1)	6 (10.3)	2 (3.7)	
None	84 (75)	50 (86.2)	34 (63.0)	
2. Which factors contributed to the above difficulties?				
Difficult airway	9 (7.8)	1 (1.7)	8 (14.3)	0.053
Laringospam	16 (13.9)	6 (10.2)	10 (17.9)	
Hemodynamic alterations	3 (2.6)	1 (1.7)	2 (3.6)	
Others	2 (1.7)	1 (1.7)	1 (1.7)	
None	85 (73.9)	50 (84.7)	35 (62.5)	

## Supplementary Materials

The following are available online at http://www.mdpi.com/2077-0383/7/11/421/s1, Table S1: EBUS-TBNA-specific questionnaires, Table S2: Sedatives/analgesic drugs and respective doses used.

## Appendix A

**Table A1:** Ramsey sedation scale (RSS).

Score	Response
Ramsey 1	Anxious, agitated, restless
Ramsey 2	Cooperative, oriented, tranquil
Ramsey 3	Responsive to commands only
Ramsey 4	Brisk response to light glabellar tap or loud auditory stimulus
Ramsey 5	Sluggish response to light glabellar tap or loud auditory stimulus
Ramsey 6	No response to light glabellar tap or loud auditory stimulus
