# Supplementary material for: Endobronchial Ultrasound under Moderate Sedation versus General Anesthesia
Source: J Clin Med. 2018 Nov 8;7(11):421. doi: 10.3390/jcm7110421 (PMC6262282; doi:10.3390/jcm7110421)
Supplement: Supplementary file 1 [file jcm-07-00421-s001.pdf]

Supplementary material

Table S1. EBUS-TBNA specific questionnaires.

**Questionnaire 1 - Knowledge and fears about the exam**				
	**Total n (%)**	**Group 1 n (%)**	**Group 2 n (%)**	***p value***
1. Have you heard about this exam?				
a) Yes	36 (31.6)	17 (29.3)	19 (33.9)	0.596
b) No	78 (68.4)	41 (70.7)	37 (66.1)	
2. Have you done this exam before?				
a) Yes	8 (7.0)	3 (5.1)	5 (8.9)	0.418
b) No	107 (93.0)	56 (94.9)	51 (91.1)	
3. What is your biggest fear regarding this exam?				
a) Shortness of breath	5 (4.3)	4 (6.8)	1 (1.8)	0.399
b) Death	3 (2.6)	2 (3.4)	1 (1.8)	
c) Results of the test	31 (27.0)	18 (30.5)	13 (23.2)	
d) Do not fall completely asleep	1 (0.9)	0	1 (1.8)	
e) Do not wake up from anesthesia	11 (9.6)	5 (8.5)	6 (10.7)	
f) None	38 (33.0)	13 (22.0)	25 (44.6)	
g) More than one	26 (22.6)	17 (28.8)	9 (16.1)	
**Questionnaire 2 - Exam satisfaction and complaints**				
1. What was the worst moment during the exam?				
a) Waiting period	14 (12.2)	6 (10.2)	8 (14.3)	0.778
b) Onset of anesthesia	16 (13.9)	8 (13.6)	8 (14.3)	
c) During anesthesia	0	0	0	
d) Wake up from anesthesia	0	0	0	
e) No unpleasant moments	85 (73.9)	45 (76.3)	40 (71.4)	
2. Would you repeat the exam?				
a) No	7 (6.1)	5 (8.5)	2 (3.6)	0.272
b) Yes	108 (93.9)	54 (91.5)	54 (96.4)	

Table S2. Sedatives/analgesics drugs and respective doses used.

**Drugs (mg)**	**Total**	**Group 1**	**Group 2**	***p value***
Alfentanyl	1.1±0.4	1.1±0.4	1.1±0.4	0.897
Propofol	332.8±164.5	389.6±157.1	273.0±151.6	<0.001
Midazolam	1.4±0.9	1.2±0.9	1.6±0.9	0.018
